# Molecular profiling and prognostic significance of gastric cancer subtypes based on extracellular vesicle-related characteristics

**DOI:** 10.3389/fonc.2026.1948199

**Published:** 2026-10-01

**Authors:** Guangyu Ding, Chenjie Xu, Xingmao Huang, Federico Maria Mongardini, Pengfei Yu, Chengkang Zhu, Hongtao Wang, Zequan Wu, Shengxin Fang, Xinyi Lei, Minji Xu, Salvatore Tolone, Ludovico Docimo, Li Min, Ling Huang

**Affiliations:** 1 Zhejiang Cancer Hospital; Hangzhou Institute of Medicine (HIM), Chinese Academy of Sciences, Hangzhou, China; 2 Beijing Friendship Hospital, Capital Medical University, Beijing, China; 3 Department of Gastroenterology, Beijing Friendship Hospital, Capital Medical University, State Key Laboratory of Digestive Health, National Clinical Research Center for Digestive Diseases, Beijing Key Laboratory for Precancerous Lesion of Digestive Diseases, Beijing, China; 4 Department of Gastrointestinal Surgery, The First Affiliated Hospital of Ningbo University, Ningbo, Zhejiang, China; 5 Division of General, Oncological, Mini-Invasive and Obesity Surgery, University of Campania “Luigi Vanvitelli”, Naples, Italy; 6 Department of Hematology, The First Affiliated Hospital of Wenzhou Medical University, Wenzhou, China

**Keywords:** caveolae-mediated endocytosis, endocytosis, extracellular vesicles, gastric cancer, immune checkpoint markers, molecular subtypes

## Abstract

**Background:**

Gastric cancer (GC) is a molecularly heterogeneous disease. This study aims to explore whether endocytosis pathway-associated genes and immune checkpoint-related features were linked to GC subtypes and prognosis.

**Methods:**

Public transcriptomic datasets from TCGA and GEO were analyzed. ssGSEA was applied to predefined endocytosis-related gene sets, and NMF was used to identify molecular subtypes. Survival, tumor microenvironment features, tumor mutation burden, microsatellite instability, and differentially expressed genes were evaluated. A three-gene prognostic model was developed and assessed through retrospective internal and external validation across public cohorts for prognostic association only.

**Results:**

Two endocytosis-related subtypes were identified. The Group 1 subtype showed higher pathway activity, more immunosuppressive microenvironmental features, higher expression of selected immune checkpoint-related markers, and poorer survival. The Group 2 subtype showed lower pathway activity and more favorable outcomes. A three-gene signature (NRP1, SERPINE1, and MISP3) stratified patients into higher-risk and lower-risk groups across retrospective cohorts. Higher-risk cases were associated with worse overall survival and enrichment of epithelial-mesenchymal transition- and immune evasion-related pathways. This retrospective cross-cohort validation suggests that the prognostic signal was not restricted to a single dataset, but it does not demonstrate prospective clinical validity, analytical validity, or readiness for clinical implementation.

**Conclusions:**

Endocytosis-related molecular patterns may be associated with distinct biological and prognostic features in GC. The identified subtypes and prognostic signature should be interpreted as exploratory, hypothesis-generating findings that warrant further prospective, functional, and assay-level validation.

## 1 Background

Gastric cancer (GC) remains a major global health burden, ranking as the fifth most common diagnosed cancer and the third leading cause of cancer-related deaths worldwide (1, 2). Despite advances in treatment, the prognosis for advanced GC is poor, largely due to its profound molecular and clinical heterogeneity (3, 4). This variability underscores the urgent need for subtype-specific biomarkers and tailored therapeutic strategies (5).

Exosomes and other extracellular vesicles (EVs) play key roles in GC progression by mediating intercellular communication within the tumor microenvironment (6–10). Their biogenesis and secretion are regulated by endocytic pathways (11, 12), which also influence immune checkpoint activity and antigen presentation (13–15). Notably, immune checkpoint inhibitors such as anti-PD-1/PD-L1 agents have shown promising yet variable responses in GC (16–20), highlighting the importance of identifying predictive biomarkers. However, the interplay between endocytic processes and immune checkpoint expression across different molecular subtypes of GC remains poorly characterized.

Although molecular classifications of GC (21–24), such as those based on angiogenesis or tight junction markers like CLDN18.2—have been proposed (25, 26), its clinical utility may be limited. Therefore, systematic profiling of endocytic pathway activity and its integration with immune checkpoint signaling may provide novel insights into GC heterogeneity and reveal new biomarkers for patient stratification.

This study explores the expression patterns of endocytic pathway-related genes and immune checkpoint markers across molecular subtypes of gastric cancer (GC). The primary objectives were to identify subtypes based on endocytic pathway activity and to examine their associations with clinical outcomes, tumor mutational burden (TMB), and microsatellite instability (MSI). In addition, we aimed to develop a hypothesis-generating prognostic model based on differentially expressed genes and to evaluate its prognostic associations through retrospective analyses of public datasets. This approach was intended to support exploratory patient stratification and to provide a basis for further investigation, rather than to establish clinical utility or guide therapeutic decision-making.

## 2 Methods

### 2.1 Data acquisition and preprocessing

Gene expression profiles and clinical information for gastric cancer (GC) were obtained from The Cancer Genome Atlas (TCGA) and Gene Expression Omnibus (GEO) databases, including the GSE34942, GSE15459, and GSE66229 cohorts. The TCGA-STAD dataset included a total of 355 tissue samples, comprising 326 primary gastric tumor samples and 29 adjacent non-tumor samples. Clinical analyses were performed on the 326 patients with primary gastric tumors. Among these patients, 205 (62.9%) were male and 121 (37.1%) were female, with a median age of 67 years (range, 35–90 years). Pathological stage was distributed as follows: Stage I, 46 patients (14.1%); Stage II, 103 patients (31.6%); Stage III, 141 patients (43.3%); and Stage IV, 36 patients (11.0%). The median follow-up time was 15.9 months (range, 0.1–124.0 months). The clinicopathological characteristics of the patients included in the final analysis are summarized in **Table 1**.

**Table 1:** Clinical characteristics of the study population.

Characteristic	Overall	Group 1	Group 2	P value
**Patients, n**	326	214	112	
**Age, years, median (IQR)**	67 (58, 72)	66 (57, 72)	68 (59, 73)	0.206
Sex				0.535
Female	121 (37.1%)	82 (38.3%)	39 (34.8%)	
Male	205 (62.9%)	132 (61.7%)	73 (65.2%)	
Pathological stage				0.13
Stage I	46 (14.1%)	25 (11.7%)	21 (18.8%)	
Stage II	103 (31.6%)	71 (33.2%)	32 (28.6%)	
Stage III	141 (43.3%)	90 (42.1%)	51 (45.5%)	
Stage IV	36 (11.0%)	28 (13.1%)	8 (7.1%)	
Pathological T category				0.217
T1	16 (4.9%)	7 (3.3%)	9 (8.0%)	
T2	66 (20.2%)	41 (19.2%)	25 (22.3%)	
T3	153 (46.9%)	105 (49.1%)	48 (42.9%)	
T4	91 (27.9%)	61 (28.5%)	30 (26.8%)	
Pathological N category				0.638
N0	104 (32.0%)	65 (30.5%)	39 (34.8%)	
N1	84 (25.8%)	59 (27.7%)	25 (22.3%)	
N2	69 (21.2%)	43 (20.2%)	26 (23.2%)	
N3	68 (20.9%)	46 (21.6%)	22 (19.6%)	
Unknown/not reported	1 (0.3%)	1 (0.5%)	0 (0.0%)	
Pathological M category				0.186
M0	303 (92.9%)	196 (91.6%)	107 (95.5%)	
M1	23 (7.1%)	18 (8.4%)	5 (4.5%)	
Outcome				0.047
Alive	194 (59.5%)	119 (55.6%)	75 (67.0%)	
Dead	132 (40.5%)	95 (44.4%)	37 (33.0%)	

The bold text indicates the names of the characteristic categories, such as “Sex” and “Pathological stage.”

RNA sequencing data in fragments per kilobase per million (FPKM) format were downloaded from the Genomic Data Commons (GDC) portal, and converted to transcripts per kilobase million (TPM) for downstream analysis. Microarray data were acquired in ‘CEL’ format and normalized using the robust multiarray averaging algorithm implemented in the ‘affy’ and ‘simpleaffy’ R packages.

### 2.2 Identification of GC molecular subtypes based on endocytic pathway activity

A gene set related to endocytosis—covering pathways of EV biogenesis, composition, and uptake—was compiled from relevant literature and molecular databases. Single-sample gene set enrichment analysis (ssGSEA) was performed using the “GSVA” R package to calculate enrichment scores representing endocytic pathway activity per sample.

Unsupervised clustering using non-negative matrix factorization (NMF) was applied to the ssGSEA-derived endocytic pathway scores to identify molecular subtypes. The resulting sample distributions were visualized using dimensionality-reduction analyses. The optimal number of clusters was determined using silhouette coefficients. NMF initially identified three groups, namely C1, C2, and C3. According to the consensus clustering results, samples in C1 and C3 were subsequently merged and defined as subtype Group 1 with high endocytic pathway activity, while samples in subtype C2 were classified as Group 2 with low endocytic pathway activity.

### 2.3 Comparative analysis between subtypes

Kaplan-Meier survival curves were generated for overall survival (OS) and disease-specific survival (DSS), compared using the log-rank test. Cox regression analysis was used to identify prognostic markers, with hazard ratios (HR) and 95% confidence intervals (CIs) calculated for each significant gene.

The tumor microenvironment (TME) was characterized using the “ESTIMATE” R package to compute immune/stromal scores and tumor purity. Somatic mutation data for STAD were downloaded from the GDC Data Portal (https://portal.gdc.cancer.gov/) using TCGAbiolinks. Mutations were annotated using maftools, and the tumor mutation burden (TMB) was calculated using the tmb function with a capture size of 50 Mb, followed by log2 transformation. Oncoplots were generated to visualize the top 30 mutated genes. MSI data were downloaded from cBioPortal and transformed by log2(MSI score). Differences in TME, TMB, and MSI among subtypes were visualized using box plots, and their impact on prognosis was analyzed in conjunction with clinical features. Functional enrichment analyses were conducted using GSEA with Hallmark gene sets, and GO/KEGG pathway analyses were performed with the “clusterProfiler” R package.

### 2.4 Differential gene expression and prognostic modeling

Differentially expressed genes (DEGs) between molecular subtypes were identified using the limma R package, with thresholds of false discovery rate (FDR) < 0.05 and |log2 fold change| > 0.585. Univariate Cox regression analysis was performed to identify DEGs associated with overall survival (OS), followed by Lasso regression for feature selection. This procedure resulted in a three-gene prognostic signature comprising NRP1, SERPINE1, and MISP3. The risk score for each patient was calculated using the following formula: risk score = 0.021 × NRP1 expression + 0.047 × SERPINE1 expression − 0.024 × MISP3 expression, where the coefficients were derived from the TCGA-STAD training cohort. Patients were stratified into high- and low-risk groups according to the median risk score within each cohort. Model performance was evaluated using Kaplan–Meier survival analysis, log-rank tests, time-dependent receiver operating characteristic (ROC) curves, and the concordance index (C-index) in the TCGA-STAD cohort and independent GEO validation cohorts.

### 2.5 Immunohistochemistry

Immunohistochemistry (IHC) was performed to explore the protein expression patterns of NRP1, SERPINE1, and MISP3 in gastric cancer (GC) tissues and matched adjacent normal tissues. A total of 20 paired GC and adjacent non-tumor tissue samples were included in this analysis. Specimens were fixed in 10% neutral-buffered formalin, embedded in paraffin, and sectioned at 4 µm. Antigen retrieval was conducted using citrate buffer (pH 6.0) in a microwave oven. Endogenous peroxidase activity was inhibited by incubating the sections in 3% hydrogen peroxide for 10 min. Following the blocking of non-specific binding with a blocking serum for 30 min, the slides were incubated with primary antibodies overnight at 4°C. Primary antibodies used were: NRP1 (1:200, 8829-MSM2-P1, Thermo Fisher Scientific), SERPINE1 (1:200, MA5-17171, Thermo Fisher Scientific), and MISP3 (1:200, PA5-59921, Thermo Fisher Scientific).

Quantitative assessment of IHC staining was performed using ImageJ software. The average optical density (AOD) of the immunostaining was measured for each specimen and used as the quantitative staining score, with higher AOD values indicating stronger protein staining. The IHC staining procedure was performed by one investigator, whereas image quantification was independently conducted by a second investigator who was blinded to sample identity and tissue grouping. The same ImageJ-based quantification procedure was applied consistently across all samples. Because image quantification was performed by a single blinded evaluator, no formal interobserver agreement assessment was conducted. As the GC tissues and adjacent non-tumor tissues were paired samples obtained from the same patients, differences in AOD values were evaluated using a two-tailed paired Student’s t-test. A P value < 0.05 was considered statistically significant.

### 2.6 Model validation

The prognostic model was internally evaluated in the TCGA-STAD cohort and externally validated in three independent GEO cohorts (GSE34942, GSE15459, and GSE66229). The regression coefficients derived from the TCGA-STAD training cohort were kept fixed and applied directly to each external cohort without coefficient re-estimation. Because gene-expression distributions differed across datasets and experimental platforms, the median risk score within each individual cohort was used as the cutoff to classify patients into high- and low-risk groups; therefore, the numerical cutoff was cohort-specific rather than fixed to the TCGA-STAD median. Kaplan–Meier survival analysis was used to assess survival differences between risk groups, and time-dependent receiver operating characteristic (ROC) curves were generated to evaluate prognostic discrimination for 1-, 3-, and 5-year overall survival. A nomogram was constructed by integrating clinical variables (e.g., age and stage) with the gene-based risk score to estimate survival probabilities at 1, 3, and 5 years. Calibration curves were used to assess agreement between predicted and observed survival outcomes.

### 2.7 Ethical statements

This study was approved by the Medical Ethics Committee of Zhejiang Cancer Hospital (Approval No. IRB-2023–724 [IIT]) and was conducted in accordance with the Declaration of Helsinki.

### 2.8 Statistical analysis

All statistical analyses were performed using R software (version 4.0.2). Group comparisons were performed using the Wilcoxon rank-sum test or two-tailed Student’s t-test, as appropriate. For the IHC analysis, GC tissues and matched adjacent non-tumor tissues from the same patients were treated as paired samples and compared using a two-tailed paired Student’s t-test. Survival differences were assessed with log-rank tests, and Cox proportional hazards models were used to compute hazard ratios (HR) and 95% confidence intervals (CI). The performance of the prognostic model was quantified using the concordance index (C-index) and area under the ROC curve (AUC). A p-value < 0.05 or FDR < 0.05 was considered statistically significant.

## 3 Results

### 3.1 Correlation of EVs pathway activity with tumor subtype, survival, and clinical parameters in GC

Based on the cohort from TCGA-STAD described in the Methods, initial exploration of endocytosis-related gene expression patterns suggested that endocytic pathway activity varied across samples with different clinical characteristics, including tumor subtype, survival status, sex, age, and disease stage (**Figure 1A**). Notably, pathways such as clathrin-mediated endocytosis and caveolae-mediated endocytosis were significantly upregulated, suggesting their prominent role in EV uptake (**Supplementary Figure S1A**). Conversely, pathways involved in EV biogenesis (e.g., ESCRT-dependent machinery) showed more modest changes. Forest plot analysis further indicated that high activity of several endocytosis-related pathways (e.g., multivesicular body formation, macropinocytosis) was significantly associated with poor prognosis (HR > 1.5) (**Figure 1B**).

**Figure 1:**
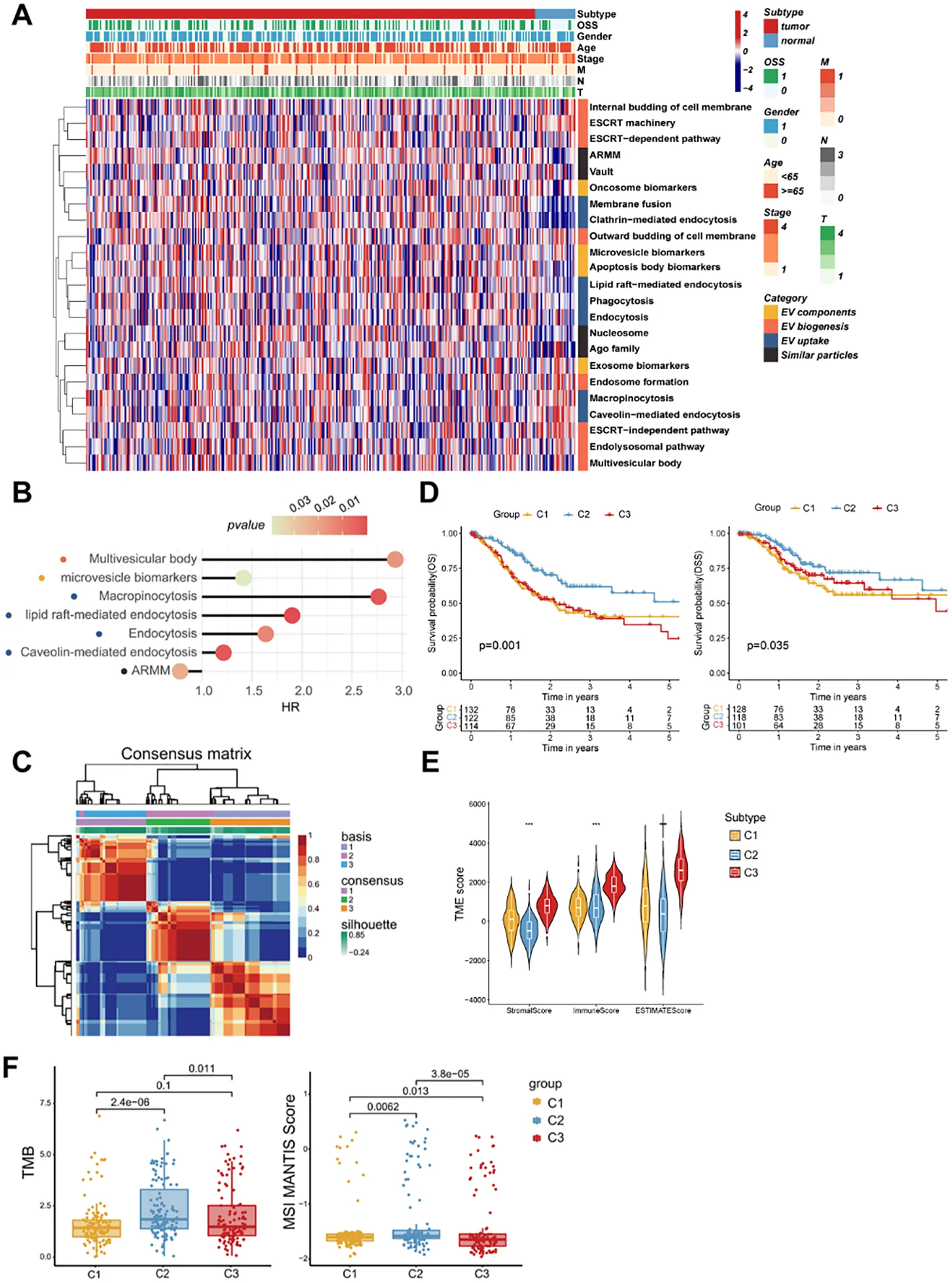
Comprehensive analysis of endocytic and extracellular vesicle-related pathway features, molecular subtypes, tumor microenvironment characteristics, and prognostic associations in GC. **(A)** Heatmap showing endocytic and extracellular vesicle (EV)-related pathway features across gastric cancer samples. **(B)** Forest plot showing the associations between selected pathway activities and overall survival; hazard ratios (HRs) and 95% confidence intervals **(CIs)** were estimated using Cox regression analysis. **(C)** Consensus matrix showing the three initial molecular clusters (C1, C2, and C3) identified by non-negative matrix factorization (NMF). **(D)** Kaplan–Meier curves comparing overall survival (OS) and disease-specific survival (DSS) among the three clusters; P values were calculated using the log-rank test. **(E)** Comparison of stromal, immune, and ESTIMATE scores among the three clusters. **(F)** Distribution of tumor mutation burden (TMB) and microsatellite instability (MSI) scores among the three clusters. Between-group comparisons were performed using the statistical procedures described in the Methods.

Unsupervised clustering of ssGSEA scores via NMF initially suggested three molecular subtypes (Groups C1-C3, **Figure 1C**), which were corroborated by distinct clustering in PCA (**Supplementary Figure S1B**). Survival analysis revealed that Group C2 had the most favorable OS and DSS, while Groups C1 and C3 exhibited similarly poor outcomes (**Figure 1D**). Evaluation of the tumor microenvironment revealed substantially elevated immune and stromal scores in Group 3 compared to the other groups (*P* < 0.001), indicating a more complex tumor microenvironment (**Figure 1E**). Given the comparable aggressive phenotypes and to enhance analytical clarity, we consolidated Groups C1 and C3 into a single subtype designated Group 1 (high endocytic pathway activity), and retained Group 2 as Group C2 (low endocytic pathway activity) for all subsequent analyses.

### 3.2 GC molecular subtypes are associated with different endocytic, immune, and metabolic pathways associated with tumor prognosis

Comparative analysis between the Group 1 and Group 2 subtypes revealed fundamental differences in their tumor microenvironment (TME) and biological functions. The Group 1 subtype exhibited significantly higher activity of ESCRT-related pathways and higher levels of exosome- and microvesicle-related molecular features, whereas these features were relatively reduced in Group 2 (**Figure 2A**). A total of 871 differentially expressed genes (DEGs) were identified between Group 1 and Group 2 subtypes (FDR < 0.05, |log_2_FC| > 0.585) (**Figure 2B**).

**Figure 2:**
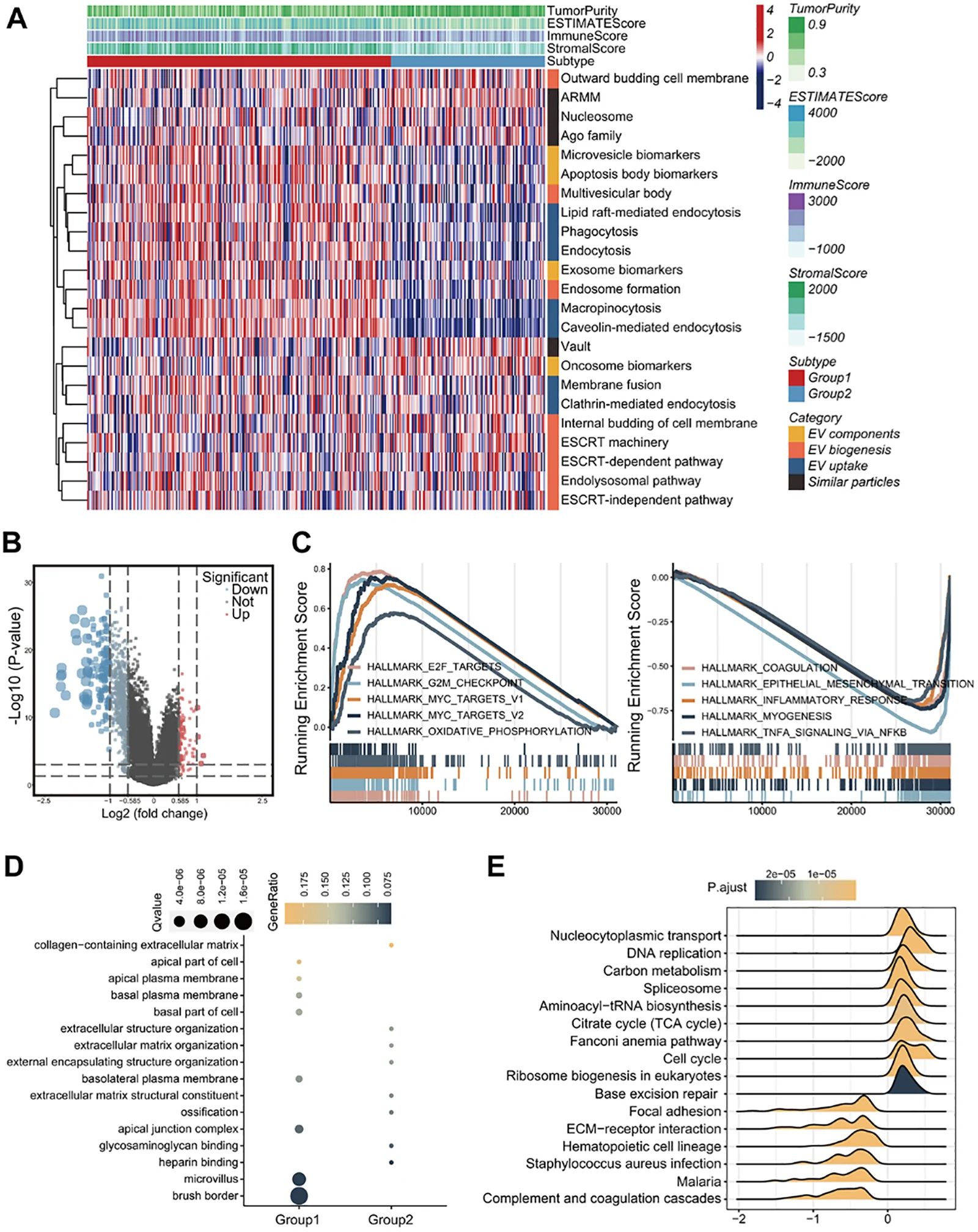
Molecular characterization of GC subtypes reveals distinct tumor microenvironment and biological pathway features. **(A)** Heatmap showing differences in biological pathway features and tumor microenvironment (TME) characteristics between Group 1 and Group 2. **(B)** Volcano plot showing differentially expressed genes (DEGs) between the two groups. DEGs were defined as false discovery rate (FDR) < 0.05 and |log2 fold change| > 0.585. **(C)** Gene set enrichment analysis (GSEA) showing representative Hallmark pathways differentially enriched between the two groups. **(D)** Bubble plot showing functional enrichment of subtype-associated genes. **(E)** Ridge plot showing enrichment-score distributions for selected biological processes and pathways.

The Group 1 subtype was characterized by significantly elevated stromal and immune scores (**Figure 1E**), suggesting a complex yet dysfunctional TME. Despite this immune infiltration, Group 1 tumors showed a strong signature of immune suppression, with significant upregulation of cell proliferation pathways (E2F targets, G2M checkpoint) and epithelial-mesenchymal transition (EMT) (**Figure 2C**). Functional enrichment analysis further indicated heightened activity in processes like nucleocytoplasmic transport and microvillus assembly (**Figures 2D, E**), consistent with a highly proliferative and invasive phenotype.

In contrast, the Group 2 subtype, while having lower overall immune scores, displayed enrichment for pathways related to heparin binding and cell-cell communication (**Figures 2D, E**), suggesting a less aggressive TME composition. Although the Group 2 subtype had a higher tumor mutational burden (TMB) and microsatellite instability (MSI) (**Figure 1F**)—features often associated with immunotherapy response—the Group 2 subtype demonstrated a slightly higher proportion of MSI-H tumors (**Supplementary Figure S2**), hinting at a complex interplay between mutagenesis and immune evasion.

### 3.3 Construction and retrospective evaluation of a three-gene prognostic risk model

To further explore the prognostic relevance of subtype-associated genes, we developed a three-gene prognostic model based on differentially expressed genes (DEGs) between Group 1 and Group 2. Univariate Cox analysis of 871 DEGs identified 378 prognosis-associated genes (**Figure 3A**). Lasso regression refined this to a parsimonious three-gene signature (NRP1, SERPINE1, MISP3) (**Figure 3B**). Using the three-gene formula described above, a risk score was calculated for each patient in the TCGA-STAD cohort, and patients were stratified into high- and low-risk groups according to the cohort-specific median risk score.

**Figure 3:**
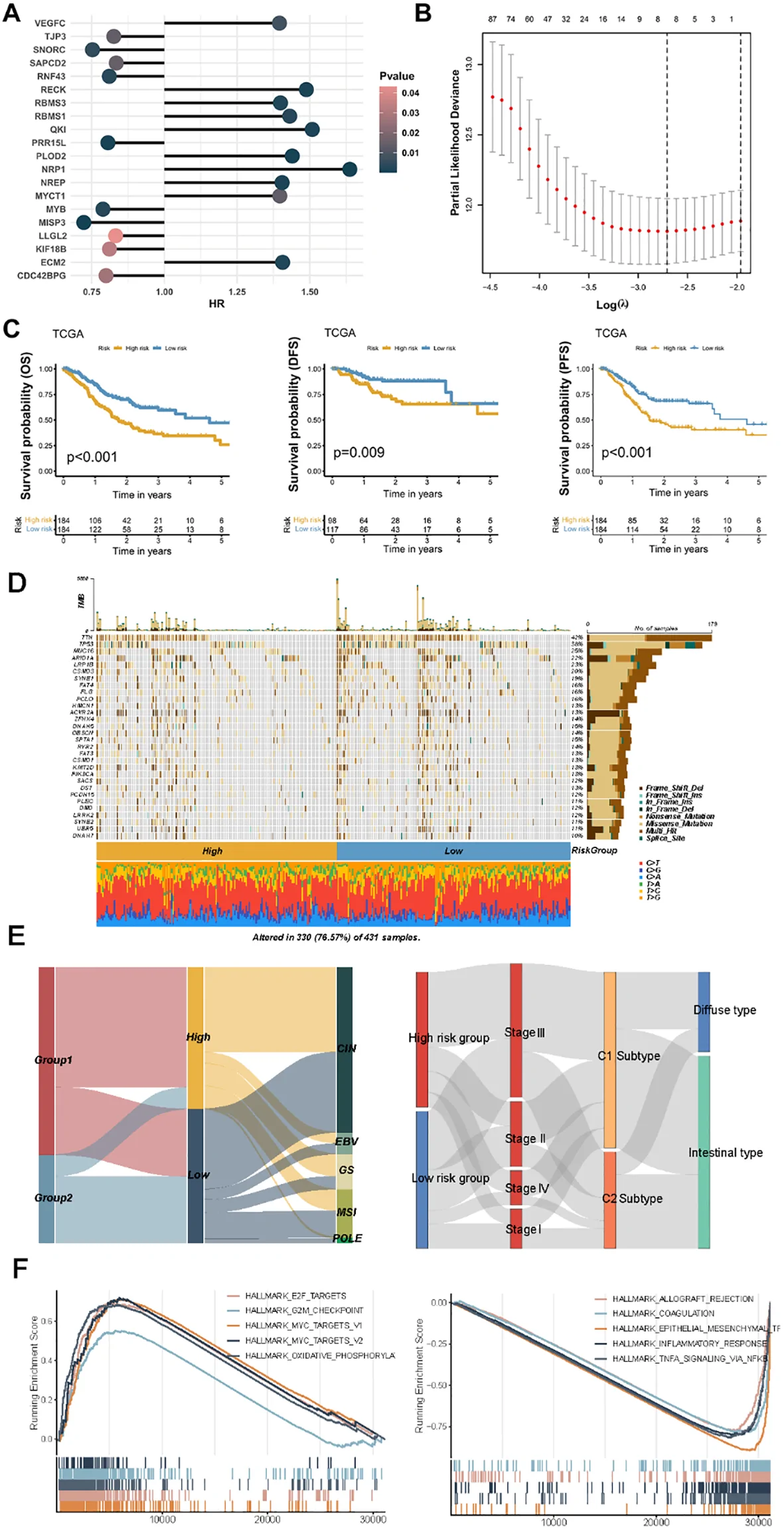
Identification of endocytic pathway genes as key biomarkers for risk stratification and prognosis in GC. **(A)** Univariate Cox regression analysis of prognosis-associated genes; hazard ratios (HRs) and 95% confidence intervals (CIs) are shown. **(B)** Lasso regression analysis used for feature selection and identification of the three-gene signature comprising NRP1, SERPINE1, and MISP3. **(C)** Kaplan–Meier curves comparing overall survival (OS), disease-free survival (DFS), and progression-free survival (PFS) between high- and low-risk groups; P values were calculated using the log-rank test. **(D)** Somatic mutation profiles of the high- and low-risk groups. **(E)** Sankey diagram showing relationships among risk groups, molecular subtypes, Lauren classification, and pathological stage. **(F)** Gene set enrichment analysis (GSEA) showing representative pathways enriched in the two risk groups.

Kaplan-Meier analysis demonstrated significantly poorer survival in the high-risk group across overall (OS), disease-free (DFS), and progression-free (PFS) survival endpoints (**Figure 3C**). Mutation profiling indicated a higher tumor mutational burden (TMB) in high-risk patients (p < 0.001), with frequent mutations in TP53, TTN, and MUC16 (**Figure 3D**) and Sankey diagram illustrated associations between endocytic pathway activity, risk stratification, and molecular subtypes, revealing a predominance of high-risk cases in aggressive CIN subtypes (**Figure 3E**). GSEA indicated upregulation of E2F targets, G2M checkpoint, MYC targets, and oxidative phosphorylation in high-risk patients, whereas low-risk patients exhibited enrichment in allograft rejection, coagulation, epithelial–mesenchymal transition (EMT), and TNFα signaling via NF-κB (**Figure 3F**).

### 3.4 Validation of the risk model in external cohorts

The risk model was evaluated in three independent GEO cohorts (GSE34942, GSE15459, and GSE66229). Patients categorized as high-risk consistently showed shorter survival than those categorized as low-risk (P < 0.05) (**Figure 4A**). Time-dependent ROC analyses yielded AUC values of approximately 0.55–0.66 at 1, 3, and 5 years across the external cohorts (**Figure 4B**), indicating modest prognostic discrimination. The risk score was positively associated with advanced tumor stage (**Figure 4D**) and remained independently associated with overall survival in multivariate Cox regression analysis (**Figure 4E**) and the complete univariate and multivariate Cox regression results are provided in **Table 2**. A nomogram incorporating the risk score and clinical variables, including age and stage, showed agreement between predicted and observed survival probabilities in retrospective analyses (**Figure 4C**). These findings provide preliminary cross-cohort support for the prognostic association of the signature but should not be interpreted as evidence of clinical utility.

**Figure 4:**
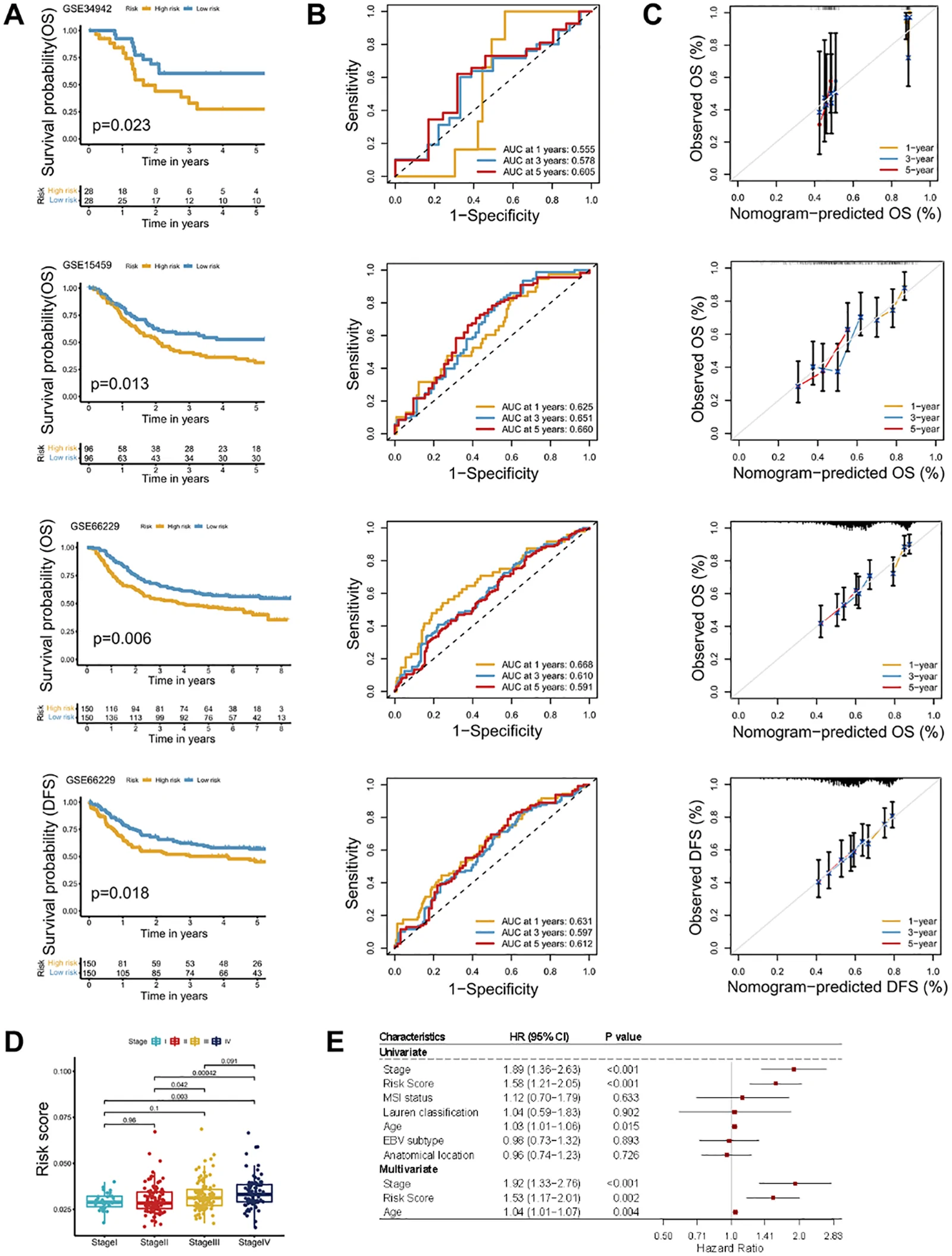
Validation of a prognostic risk model for overall survival in GC across multiple datasets. **(A)** Kaplan–Meier curves comparing overall survival between high- and low-risk groups; P values were calculated using the log-rank test. **(B)** Time-dependent receiver operating characteristic (ROC) curves evaluating prognostic discrimination at 1, 3, and 5 years; corresponding areas under the curve (AUCs) are shown. **(C)** Calibration plots comparing predicted and observed overall survival probabilities at 1, 3, and 5 years. **(D)** Distribution of risk scores across pathological stages. **(E)** Univariate and multivariate Cox regression analyses of overall survival according to clinicopathological variables and risk score.

**Table 2:** Univariate and multivariate Cox regression analyses of overall survival in the TCGA-STAD cohort.

Univariate analysis		
Variable	Univariate HR (95% CI)	P value
Age	1.021 (1.004-1.039)	0.017
Gender	1.428 (0.978-2.084)	0.065
Pathological stage	1.603 (1.292-1.988)	<0.001
T category	1.289 (1.034-1.607)	0.024
N category	1.358 (1.161-1.589)	<0.001
M category	2.105 (1.159-3.823)	0.015
Risk score	1.593 (1.310-1.937)	<0.001
Multivariate analysis		
Variable	Multivariate HR (95% CI)	P value
Age	1.031 (1.012-1.051)	0.001
Pathological stage	1.393 (0.940-2.064)	0.099
T category	1.031 (0.767-1.385)	0.84
N category	1.136 (0.915-1.412)	0.249
M category	1.630 (0.766-3.469)	0.204
Risk score	1.517 (1.238-1.859)	<0.001

### 3.5 Correlation of risk score with clinicopathological variables

Subgroup analyses were performed to further explore the association between the risk score and overall survival across different clinicopathological strata. Patients aged ≥65 or <65, both males and females, and those with early-stage (I-II) tumors in the high-risk group had significantly worse OS. While the difference was not significant in advanced-stage (III-IV) patients, likely due to the universally poor prognosis, high-risk patients within specific T3-4, N0, and M0 categories still showed significantly worse outcomes (**Figure 5A**). The nomogram effectively integrated age and risk score to predict survival probability at 1, 3, and 5 years (**Figures 5B, C**; **Supplementary Figure S3**).

**Figure 5:**
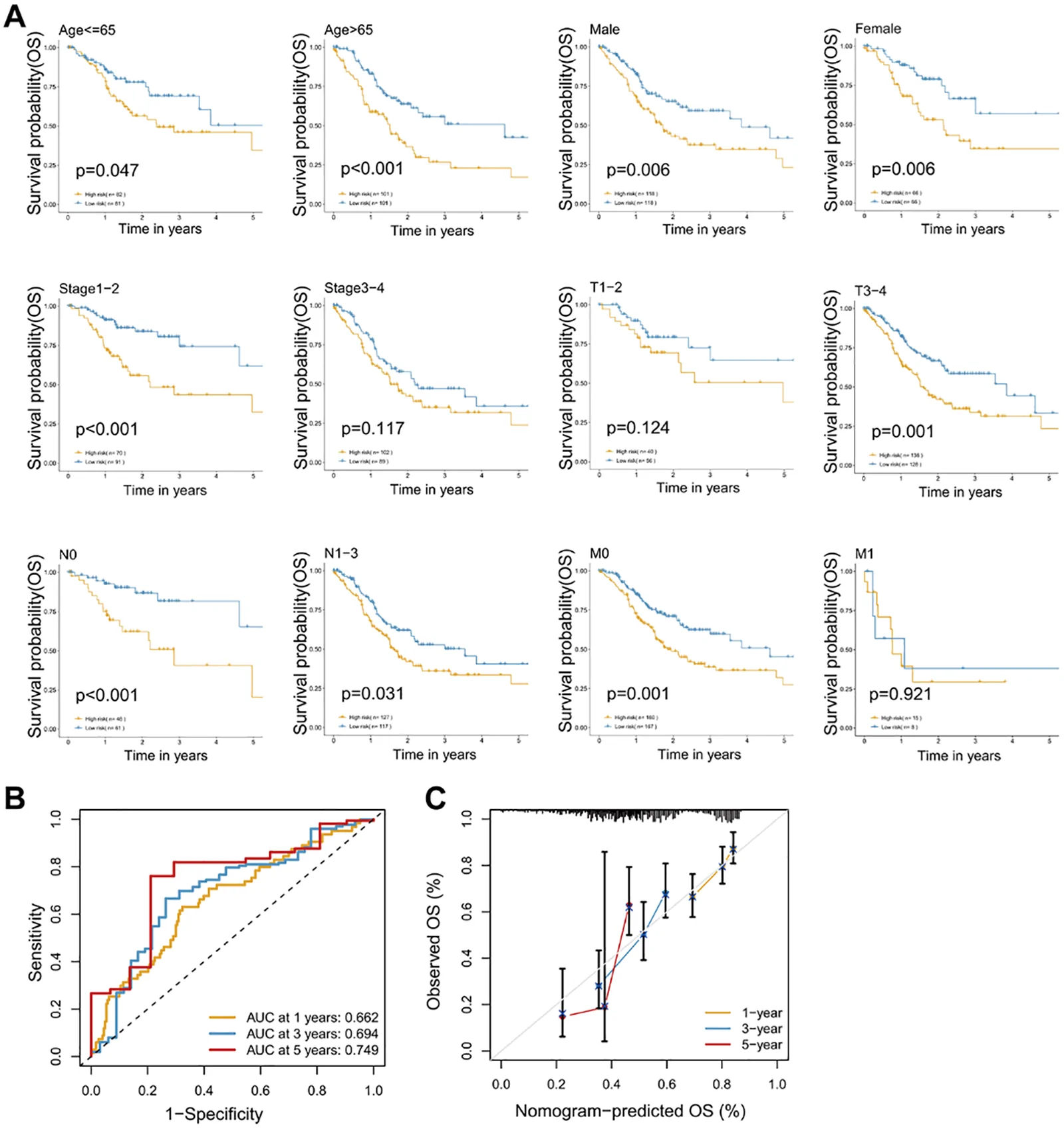
Prognostic impact of age and tumor staging on survival in GC. **(A)** Kaplan–Meier curves for by age (<65 vs. ≥65 years), sex (male vs. female), pathological stage (I–II vs. III–IV), T category (T1–2 vs. T3–4), N category (N0 vs. N1–3), and M category (M0 vs. M1). P values were calculated using the log-rank test. **(B)** Time-dependent receiver operating characteristic (ROC) curves for 1-, 3-, and 5-year OS. **(C)** Calibration curves comparing predicted and observed OS probabilities at 1, 3, and 5 years.

#### 3.5.1 Molecular characteristics of NRP1, SERPINE1, and MISP3

Expression of the model’s genes was strongly associated with risk and disease progression. NRP1 and SERPINE1 were significantly upregulated in high-risk patients and positively correlated with risk score (**Figure 6A**). Their expression increased with advancing tumor stage and T category (**Figures 6B, C**). Conversely, MISP3 was downregulated in high-risk patients, showed a negative correlation with risk score, and was expressed at higher levels in early-stage (I-II) and T1-T2 tumors (**Figures 6A–C**).

**Figure 6:**
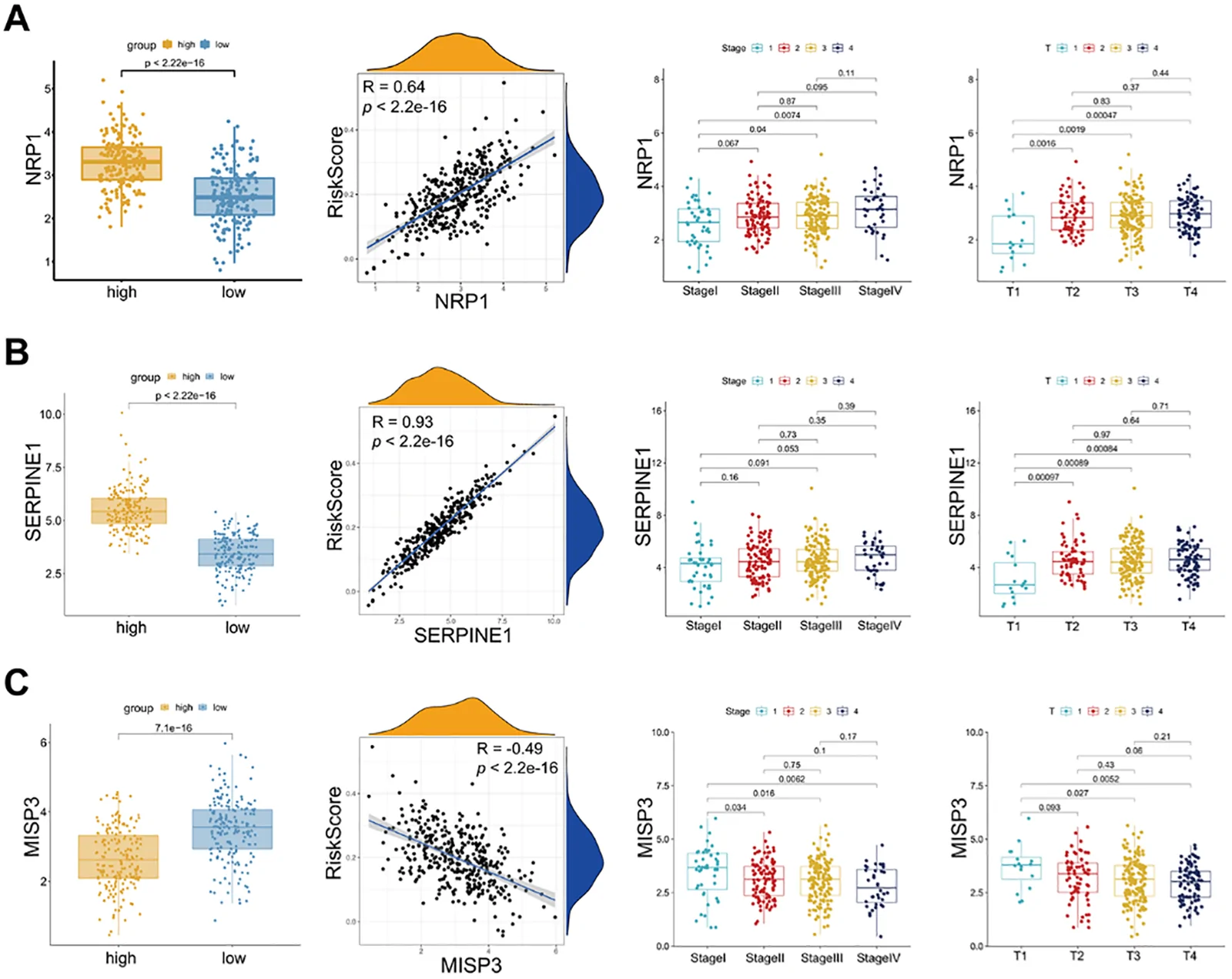
Expression analysis of key genes across risk groups and tumor stages in GC. **(A)** Comparison of NRP1, SERPINE1, and MISP3 expression between high- and low-risk groups. **(B)** Expression of the three genes across pathological stages I–IV. **(C)** Expression of the three genes across T categories T1–T4.

#### 3.5.2 Immune checkpoint profiling and therapeutic implications in GC

Given the central role of the immune microenvironment, expression of immune checkpoint molecules was profiled. The Group 1 subtype/high-risk group exhibited significantly higher expression of multiple inhibitory checkpoints including HAVCR2, TGFβ1R, PDCD1LG2, and CSF1R (**Figure 7A**), and lower predicted anti-PD1 response scores (**Figure 7B**), reinforcing its immunosuppressive characteristics. Although risk scores between immunotherapy responders and non-responders did not differ significantly (*P* = 0.066, **Figure 7C**), analysis of immunophenoscore (IPS) suggested potential differential responsiveness to CTLA-4 blockade between subtypes (**Figure 7D**). Further supporting immune dysregulation, the Group 1 subtype showed altered expression of HLA genes (**Figure 7E**) and significant differences in exosome-related functional pathways (**Figure 7F**). Correlation analysis positioned NRP1 and SERPINE1 within a network of exosome-related processes, while MISP3 was negatively correlated with such features (**Figure 7G**). Further correlation analysis demonstrated distinct associations between the signature genes and exosome-related functional pathways, with NRP1 and SERPINE1 showing generally positive correlations with exosome-associated processes, while MISP3 exhibited inverse or relatively weak associations (**Figure 7H**).

**Figure 7:**
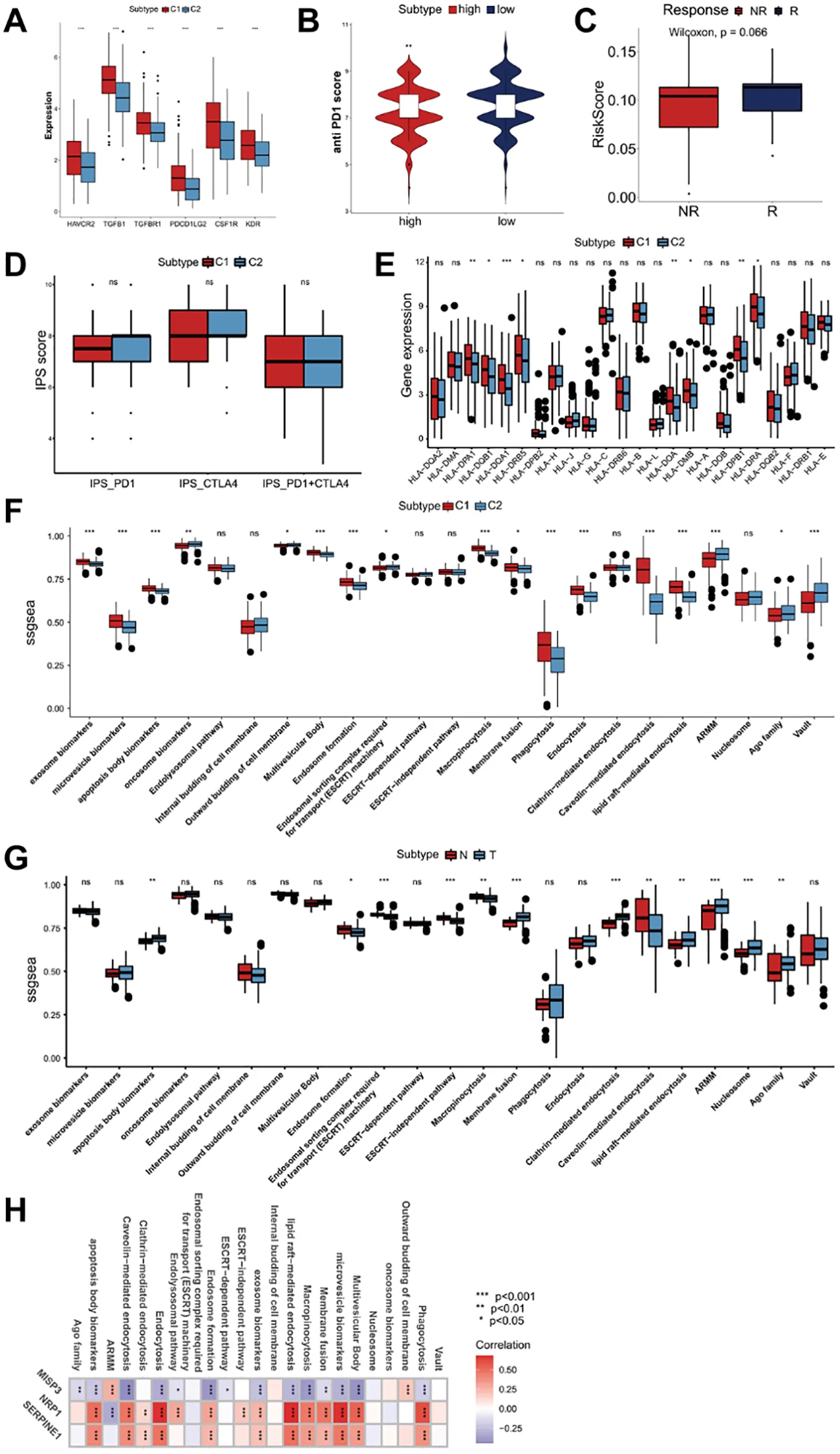
Immunotherapy response and endocytic pathway activity correlated with GC subtypes and risk scores. **(A)** Expression of selected immune checkpoint-related genes in Group 1 and Group 2. **(B)** Predicted anti-PD-1 response scores in the high- and low-risk groups. **(C)** Comparison of risk scores between immunotherapy responders and non-responders; the between-group difference was not statistically significant (P = 0.066). **(D)** Immunophenoscore (IPS) comparisons for PD-1, CTLA-4, and combined PD-1/CTLA-4-related categories between Group 1 and Group 2. **(E)** Expression of selected human leukocyte antigen (HLA) genes in Group 1 and Group 2 in the TCGA-STAD cohort. **(F)** Single-sample gene set enrichment analysis (ssGSEA) scores for extracellular vesicle-related functional pathways in Group 1 and Group 2. **(G)** Comparison of corresponding pathway scores between gastric cancer and adjacent non-tumor tissues. **(H)** Correlations between the three signature genes and extracellular vesicle-related pathway scores. Data are presented as mean ± SD where applicable. *P < 0.05, **P < 0.01, ***P < 0.001.

#### 3.5.3 Protein-level validation of NRP1, SERPINE1, and MISP3 in GC tissues

Protein expression of the three signature genes was validated using immunohistochemistry (IHC). Consistent with transcriptomic findings, NRP1 and SERPINE1 protein levels were significantly elevated in GC tissues compared to adjacent normal tissues. In contrast, MISP3 protein expression was markedly reduced in tumor samples (**Figures 8A–C**), supporting their roles in GC pathogenesis and underscoring the robustness of the molecular signature.

**Figure 8:**
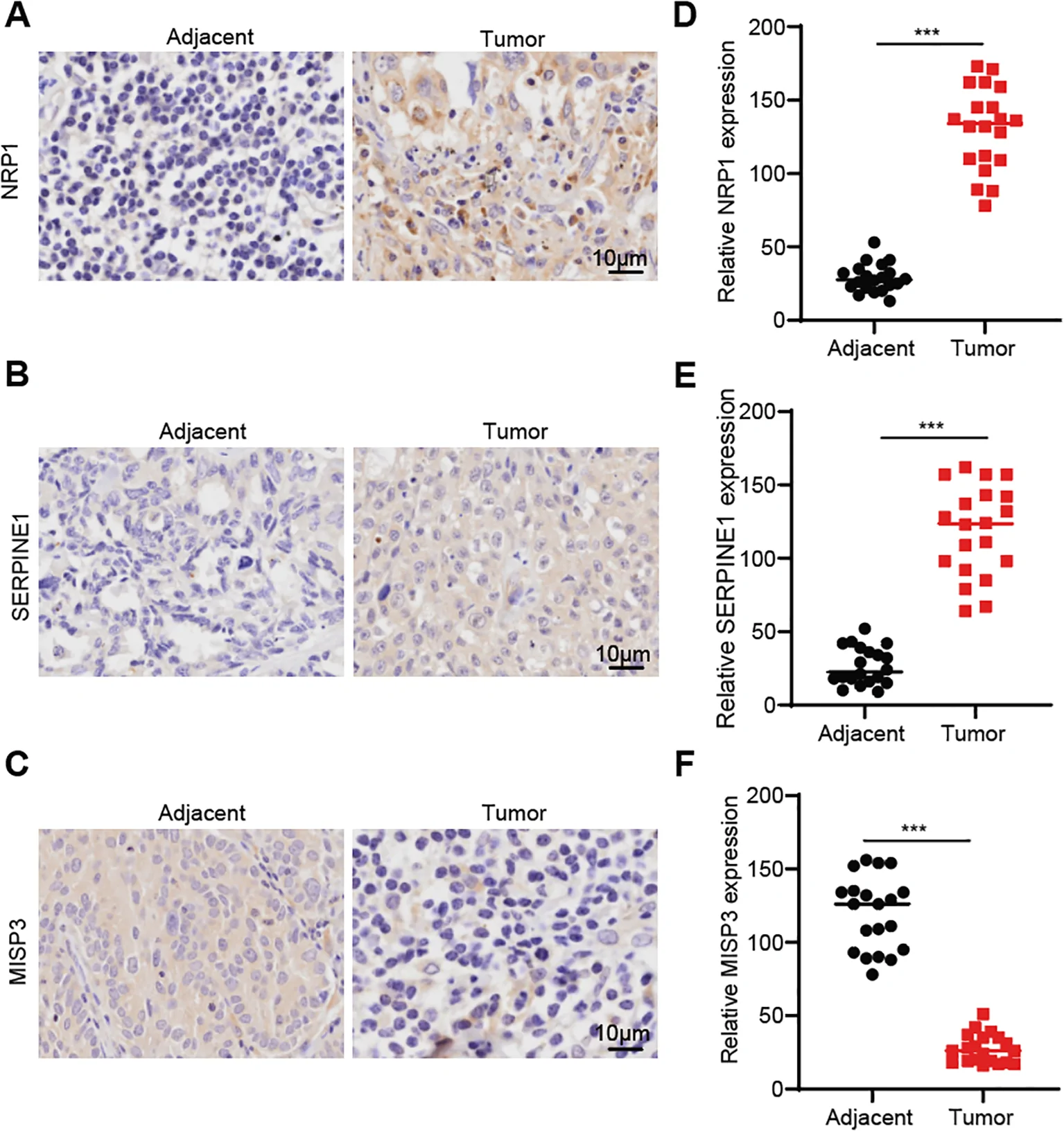
Expression of NRP1, SERPINE1, and MISP3 in GC tissues **(A–C)** Representative IHC images showing NRP1, SERPINE1, and MISP3 staining in GC tissues and matched adjacent non-tumor tissues. Scale bar = 10 µm. **(D–F)** ImageJ-based quantification of the average optical density (AOD) of NRP1, SERPINE1, and MISP3 staining in 20 paired specimens. Data are presented as mean ± SD (n = 20 pairs). Statistical comparisons between paired GC and adjacent non-tumor tissues were performed using a two-tailed paired Student’s t-test. *P < 0.05, **P < 0.01, ***P < 0.001.

## 4 Discussion

This study suggests that endocytic pathway activity may be associated with variation in the molecular and immunological landscape of gastric cancer (GC). Through retrospective bioinformatics analyses, supported by exploratory immunohistochemical assessment, we identified two subtypes with distinct characteristics: a Group 1 subtype associated with higher endocytic pathway activity, increased expression of selected immune checkpoint-related markers, and poorer overall survival, and a Group 2 subtype associated with lower pathway activity and more favorable outcomes. These findings provide exploratory insight into GC heterogeneity, but they should not be interpreted as establishing clinically actionable disease categories.

Subtype Group 1 exhibited elevated expression of immune checkpoint markers, including HAVCR2, TGFβ1R, and PDCD1LG2, alongside increased endocytic pathway activity, which correlated with poorer OS. This subtype also demonstrated a higher TMB and a pronounced EMT phenotype, which has been clearly demonstrated in previous studies to be associated with the link between EMT, TGF-β signaling pathway, and immune suppression and poor-prognosis GC subtypes (27, 28), suggesting a more aggressive tumor biology. In contrast, subtype Group 2, characterized by lower stromal and immune cell infiltration and lower endocytic pathway activity, exhibited better OS and a less aggressive clinical course. These observations highlight the dual role of endocytic pathways in promoting and suppressing tumor progression depending on the context. These observations suggest that the Group 1 subtype may represent a more aggressive biological state.

Building on this subtyping, we developed a three-gene prognostic signature comprising NRP1, SERPINE1, and MISP3. The signature stratified patients into higher- and lower-risk groups across multiple retrospective cohorts. Higher-risk patients showed poorer overall survival and distinct molecular features, including differences in mutation burden and pathways related to tumor progression and immune regulation. NRP1, a well-known regulator of angiogenesis and immune modulation, was significantly associated with poor prognosis (29–32). SERPINE1, which is involved in ECM remodeling (33–36), and MISP3 a lesser-known gene in cancer biology (37), also contributed to the risk stratification, indicating the potential complexity of the mechanisms driving GC progression.

The present retrospective cross-cohort validation provides preliminary evidence that the prognostic association of the three-gene signature is reproducible across independent datasets. Nevertheless, its discriminatory performance remains limited, and the current analyses do not establish prospective clinical validity, analytical validity, or incremental benefit beyond established clinicopathological factors. Accordingly, the signature should be regarded as an exploratory, hypothesis-generating model that warrants further prospective validation, standardized assay development, and functional investigation before any consideration of clinical implementation.

Several limitations must be acknowledged. First, this is an exploratory, hypothesis-generating bioinformatics study based on retrospective TCGA and GEO data. Despite multi-cohort validation and preliminary IHC verification, our findings only reflect potential molecular correlations rather than definitive causal relationships, and the identified endocytosis-related subtypes and three-gene signature remain testable hypotheses awaiting rigorous experimental and prospective clinical validation. Second, public database data has inherent flaws, including inconsistent sample collection and pathological evaluation criteria, incomplete follow-up, and small subcohort sample sizes, which may reduce the statistical power and generalizability of our results. Third, the specific functional mechanisms by which NRP1, SERPINE1 and MISP3 regulate endocytic pathway activity, immune checkpoint expression and immune evasion in GC remain unelucidated and need to be verified by *in vitro* and *in vivo* experiments. Fourth, this study only analyzed transcriptomic data without integrating multi-omics data such as proteomics and spatial transcriptomics, which limits the comprehensive characterization of the molecular features of endocytosis-related GC subtypes. Future studies should focus on prospective validation, formal assay evaluation, and mechanistic experiments to determine whether the identified subtypes and the three-gene signature have reproducible biological and clinical relevance. Such work will be necessary before any claims regarding clinical applicability or treatment stratification can be supported.

In conclusion, this study suggests that endocytic pathway activity may be associated with distinct molecular, immunological, and prognostic patterns in gastric cancer. We identified two endocytosis-related subtypes and developed a three-gene prognostic signature that showed prognostic associations across retrospective internal and external public cohorts. These findings should be regarded as exploratory and hypothesis-generating. While they support further investigation of endocytic pathway-related biology in GC, they do not establish clinical utility, assay robustness, treatment-selection value, or causal mechanisms. Rather, they provide a rationale for future prospective, functional, and analytical validation studies.

## Data Availability

The original contributions presented in the study are included in the article/**Supplementary Material**. Further inquiries can be directed to the corresponding authors.
