# Supplementary material for: Molecular profiling and prognostic significance of gastric cancer subtypes based on extracellular vesicle-related characteristics
Source: Front Oncol. 2026 Oct 1;16:1948199. doi: 10.3389/fonc.2026.1948199 (PMC13669850; doi:10.3389/fonc.2026.1948199)
Supplement: Supplementary file 1 [file Supplementaryfile1.docx]

**Supplementary materials**

**Figure S1. Extracellular vesicle-related processes and subtype separation.** (A) Systematic profiling of extracellular vesicle-related biological processes. The x-axis represents the log_2_ fold change (Log_2_ FC). The y-axis represents the negative logarithm of the P value (−Log_10_ P). Horizontal dashed line: −Log_10_ P = 1.3 (*P* = 0.05). Vertical dashed lines: Log_2_ FC = ± 0.05. Functionally annotated processes are color-coded. (B) Principal component analysis (PCA) of samples based on V1 and V2 profiles. The distinct separation among groups in PCA space supports the previously identified molecular subtypes derived from unsupervised NMF clustering of ssGSEA scores.

**Figure S2. Proportional distribution of molecular subtypes across Group 1 and Group 2.** Stacked bar plot comparing the relative abundance of three molecular subtypes—MSI-H (microsatellite instability-high), MSI-L (microsatellite instability-low), and MSS (microsatellite stable)—between Group 1 and Group 2.

**Figure S3. Prognostic nomogram of overall survival based on age and risk score.** The point assignment for age shows that patients aged ≥65 receive higher risk points. A nonlinear relationship between the continuous risk score and assigned points is depicted by a blue curve, with a grey band indicating variability.
